# Microstructural Features, Defects, and Corrosion Behaviour of 316L Stainless Steel Clads Deposited on Wrought Material by Powder- and Laser-Based Direct Energy Deposition with Relevance to Repair Applications

**DOI:** 10.3390/ma15207181

**Published:** 2022-10-14

**Authors:** Reynier I. Revilla, Iris De Graeve

**Affiliations:** Department of Materials and Chemistry (MACH), Research Group of Electrochemical and Surface Engineering, Vrije Universiteit Brussel (VUB), Pleinlaan 2, 1050 Brussels, Belgium

**Keywords:** 316L stainless steel, corrosion, defects, laser metal deposition, single clads

## Abstract

This work analyses the microstructural defects and the corrosion behaviour of 316L stainless steel clads deposited by laser metal deposition on wrought conventional material, which is a highly relevant system for repair applications. The different defects and microstructural features found in these systems were identified and analysed from a perspective relevant to the corrosion performance of these materials. The role of these features and defects on the corrosion process was evaluated by exposure of the samples to corrosive media and further examination of the corrosion morphology. The heat-affected zone, located on the wrought base material in close vicinity of the deposited clad, was identified to be the primary contributor to the corrosion activity of the system due to the large depletion of alloying elements in this region, which significantly decreased its pitting resistance. Alongside the heat-affected zones, relatively small (<30 µm in diameter) partially un-melted powder particles scattered across the surface of the clad were systematically identified as corrosion initiation spots, possibly due to their relatively high surface energy and therefore high reactivity compared to larger powder particles. This work highlights the need for more investigations on as-built surfaces of additively manufactured parts to better explore/understand the performance of the materials closer to their final applications. It demonstrates that the surface defects resulting from the additive manufacturing process, rather than the presence of the refined sub-granular cellular structure (as highlighted in previous works), play the predominant role in the corrosion behaviour of the system.

## 1. Introduction

Metal additive manufacturing (MAM), one of the enabling technologies for Industry 4.0 [1], is based on the production of complex multifunctional metal parts in a layer-by-layer fashion. MAM enables near net-shape manufacturing of geometrically complex parts (such as pieces with undercuts, cavities, and lattice structures), which has increased its potential application in several industries. Currently, many metals and alloys can be processed by MAM. Amongst these alloys, 316L austenitic stainless steel (SS) is of great interest due to its numerous industrial applications.

MAM processes are known to promote the formation of a highly refined microstructure with unique directional growth features far from equilibrium [2,3]. This is mainly due to the rapid solidification associated with these processes. The microstructure of additively manufactured (AM) 316L SS is characterised by a fine and interconnected network of sub-granular cells with their border enriched in alloying elements [2,3,4,5,6,7,8,9,10]. This distinctive microstructure is known to greatly influence the corrosion performance of these materials [11,12,13]. However, most of the works in the literature dedicated to investigating the corrosion behavior of AM 316L SS specimens have been conducted on parts fabricated by laser-powder bed fusion (L-PBF) [10,14,15,16,17,18,19,20,21,22,23,24,25,26,27,28]. The reason for this is because the L-PBF process allows the fabrication of pieces with very high structural complexity and a high level of precision. On the other hand, very limited research has been conducted on studying the corrosion behaviour of 316L specimens fabricated using other MAM techniques, such as direct energy deposition (DED), and specifically powder-based DED—i.e., laser metal deposition (LMD) [10,29,30,31].

Metallic parts prepared by the LMD process present a more limited shape complexity when compared to L-PBF. However, LMD allows for a relatively higher deposition rate, and it can be used on existing parts of arbitrary geometry, which makes it a favoured technology for repair applications [32,33,34,35]. Nevertheless, even though LMD-prepared 316L SS is highly relevant for industrial/repair applications, not only are the studies on the corrosion performance of these specimens very limited, but to the best of our knowledge no published work to date has focused on analysing the corrosion behaviour of LMD-repaired 316L SS parts. Therefore, this work presents the first efforts in this regard. Moreover, most works dedicated to corrosion studies of AM 316L SS are carried out on polished specimens fabricated by L-PBF to evaluate the influence of the internal microstructure on the corrosion performance of this material, while ignoring the outer-surface structure and defects. This work intends to tackle this issue by evaluating the as-built condition of the specimens. Furthermore, the few works that can be found in the literature assessing the corrosion performance of repaired metal pieces evaluate the corrosion behaviour of the base material and the deposited clads separately [36,37]. This work, on the other hand, analyses for the first time the corrosion performance of the combined repaired system, consisting of a wrought 316L base and the DED-deposited 316L clads, emphasising the role of defects and microstructural features.

In this study, microstructural features as well as the corrosion performance of 316L SS clads deposited on wrought 316L base material by using LMD were analysed, since this is a highly relevant system for repair applications. The objective of this work was to identify and characterise the different defects and microstructural features present on 316L SS clads deposited by powder- and laser-based DED, and to determine their role in the corrosion performance of the clads. The role of the different types of defects and microstructural features in the corrosion process was evaluated by exposing the samples to corrosive media, followed by a detailed examination of the corrosion morphology using scanning electron microscopy.

## 2. Experimental Section

**Figure 1:**
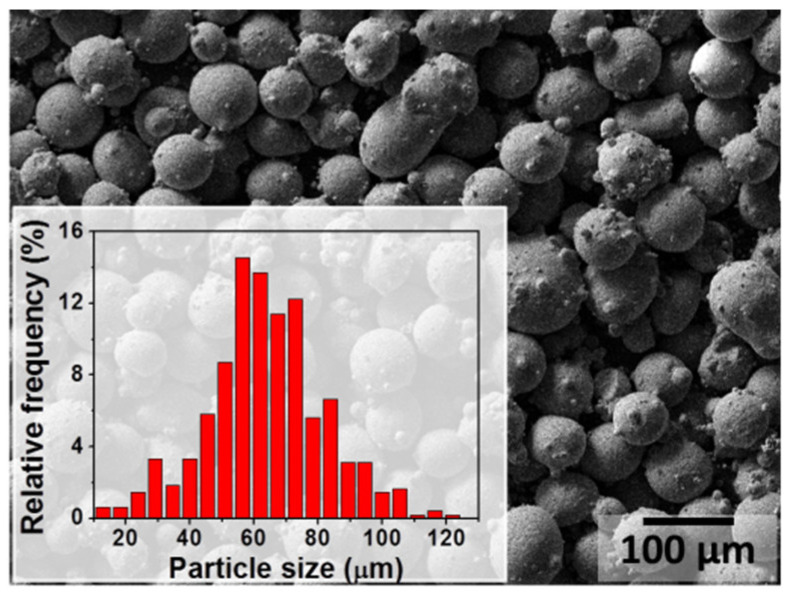
Secondary electron image of the 316L stainless steel powder used to print the samples together with the particle diameter distribution.

**Figure 2:**
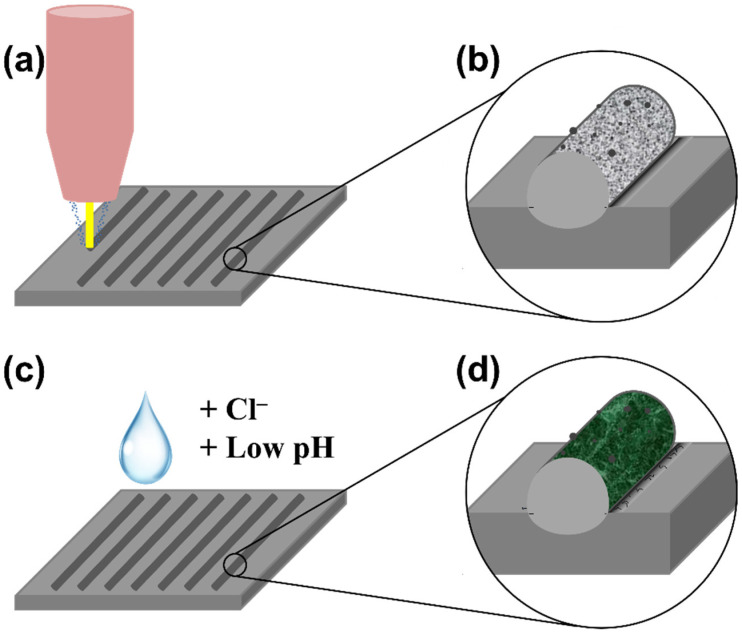
Schematic representing the workflow of the current study (from step a to d). (**a**) Printing of several 316L SS clads by LMD on wrought 316L base plate. (**b**) Analysis of microstructural features and defects. (**c**) Exposure of the samples to corrosive electrolyte (containing Cl^–^ and Cl^–^ + low pH). (**d**) Corrosion morphology analysis to determine the role of the different defects and microstructural features.

For this study, gas atomised 316L SS powder with an average particle diameter of 63 µm was utilised (see micrograph of the powder particles together with the particle size distribution in Figure 1). The nominal chemical composition of austenitic 316L SS is shown in Table 1. The samples were fabricated using an in-house hybrid laser metal deposition and high-speed milling machine (MiCLAD) of the Vrije Universiteit Brussel (VUB) [38]. During LMD, the laser beam melts the powder fed by a coaxial nozzle directly on the metallic substrate, forming a deposit that is fusion-bonded to the substrate. Several clads were printed on a 316L SS base plate by LMD using several sets of process parameters. The laser was focused on the surface of the wrought 316L SS substrate. The samples were fabricated using laser power ranging from 200 to 360 W, scanning speeds from 500 to 1200 mm/min, carrier gas flow rate of 2 to 5 NL/min, and argon as shielding gas with a flow rate of 1 to 2.5 NL/min. No post-heat treatment was applied on the samples. The workflow of the current study is schematically represented in Figure 2. Cross sections of the deposits were mechanically ground and polished, finishing with 0.04 µm standard colloidal silica suspension (OP–S). The microstructures of the samples were characterised by means of field-emission scanning electron microscopy (FE–SEM) combined with energy dispersive X-ray spectroscopy (EDX). A FE–SEM JEOL JSM–7100F was used, operating at 15 kV acceleration voltage, 5 pA probe current, and a working distance of 10 mm. To highlight the microstructural features, some samples were etched in Glyceregia reagent (15 mL glycerol, 15 mL HCl, and 5 mL HNO_3_). Corrosion tests were carried out by immersing the samples for 1 week in 3.5 wt.% NaCl or in 3.5 wt.% NaCl with pH adjusted to 2. The corrosion morphology was then analysed by FE–SEM.

## 3. Results and Discussion

### 3.1. Microstructure, Special Features, and Defects

**Figure 3:**
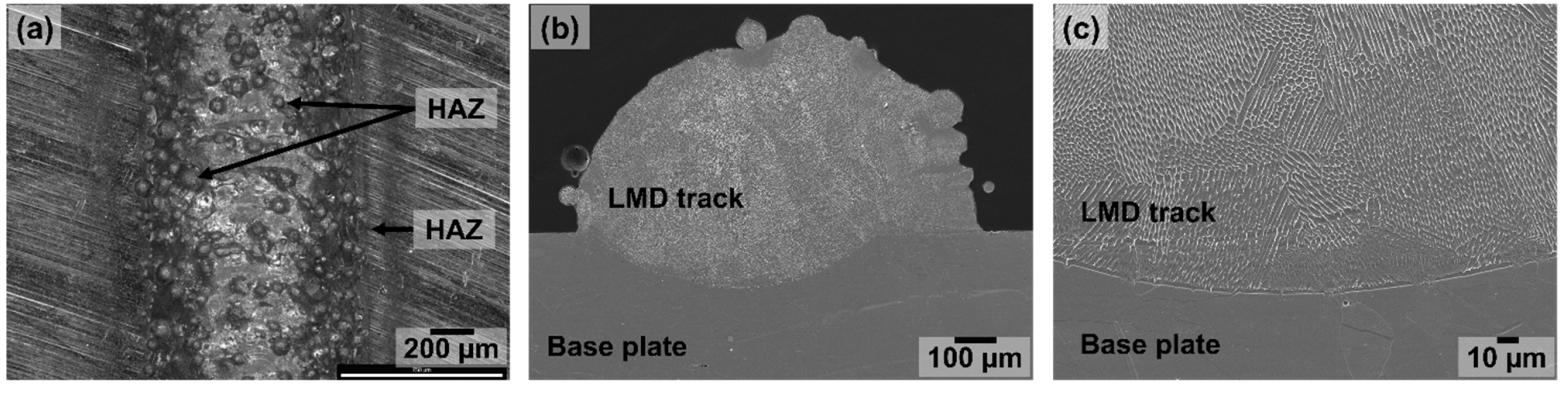
(**a**) Optical image of the top view of a typical printed clad in which partially un-melted powder particles and the heat-affected zone (HAZ) can be seen. Secondary electron images of (**b**) the cross section of a typical printed clad, and (**c**) high resolution image of a region around the interface between the printed clad and the base plate.

Figure 3a,b show representative images of the top view and the cross-section of a typical printed clad, respectively. Features such as partially un-melted metal powder particles on the surface of the clads and heat-affected zones parallel to the printed clads (on the base plate) could be easily identified on all the samples independently of the set of process parameters used. Even though the specific combination of process parameters influenced variables such as the density of partially un-melted powder particles per surface area, the same type of features and defects were present in all the printed clads. The specific relation between the manufacturing parameters and the microstructure and morphology of the clads will be discussed in a future work. The study presented in this work focused on identifying the different types of defects that can exist when depositing 316L SS on a wrought plate by LMD and their role in the corrosion of these systems (LMD-deposited clads on wrought 316L SS).

### 3.2. Microstructure

Figure 3b,c show the microstructure of the LMD-prepared clads. In these figures, the deposited clad and the base plate can be observed. A well-defined interface between the LMD-prepared clad and the conventional 316L SS base plate can be seen in Figure 3c. While the base plate presents polygonal-shaped austenitic grains (distinctive of wrought 316L SS), a sub-granular network of cells with etch-resistant borders can be seen in the LMD part. This special, sub-granular structure has already been described in several studies [2,3,4,5,6,7,8,9,10]. These cells, elongated in the direction of the thermal gradients, are characteristic of L-PBF-prepared 316L SS, as well as LMD-prepared specimens [10]. The borders of these cells were shown in earlier works to be enriched in alloying elements Cr, Ni, Mo, Mn, and Si [10]. Previous studies have shown that the formation of this sub-granular cellular network seems to play an important role in the increased pitting corrosion resistance of these additively manufactured materials compared to the conventional alloy [10,26,27,28]. The average cell diameter calculated for these printed clads ranged from 1.6 to 3.9 µm.

### 3.3. Heat-Affected Zone (HAZ) and Thermal Oxides

**Figure 4:**
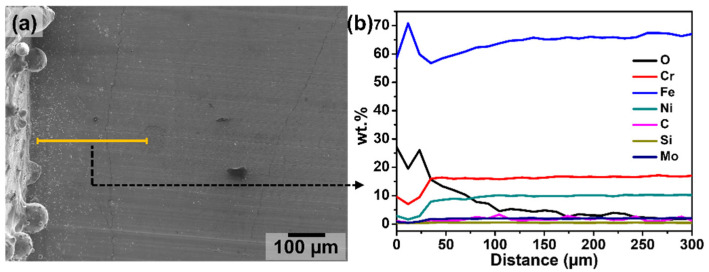
(**a**) Secondary electron image of a region around the HAZ, and (**b**) energy dispersive X-ray (EDX) composition analysis of a line scanned across the HAZ.

The heat-affected zones on the base plate in the vicinity of the printed clads were analysed by EDX. Figure 4 shows a representative example of an elemental analysis of a line scanned across the HAZ. These HAZ regions were characterised by a large depletion of Cr (7–13 wt.%), Ni (2–6 wt.%), and Mo (0.4–0.9 wt.%). The amount of Si was also slightly lower in these regions compared to the rest of the base plate. Cr and Mo are known to play a very important role in the corrosion resistance of stainless steels. A decrease in Cr and Mo denotes a decrease in the pitting resistance equivalent number (PREN = 1×%Cr + 3.3×%Mo + 16×%N [39]). The value of PREN calculated in the wrought base plate decreased from approximately 22.8 to values around 8 to 12 in the region adjacent to the printed clads (within 30 to 40 µm from the clads). The depletion of alloying elements in the HAZ is a known issue from welding applications and has been discussed in earlier studies [40,41]. 

The specific elemental composition within the HAZ varied along each deposited clad. However, in general, a depletion of Cr, Ni, Mo, and Si in these zones was always detected for all the printed clads. Since variations in the exact chemical composition of the HAZ were even seen along one single clad, no relationship could be established between the specific set of process parameters and the elemental composition of the HAZ. Nevertheless, the width of the HAZ was observed to depend on the specific set of process parameters, as also shown in previous works [32,42,43]. The greater the energy input into the material, the more extended the HAZ would be.

**Figure 5:**
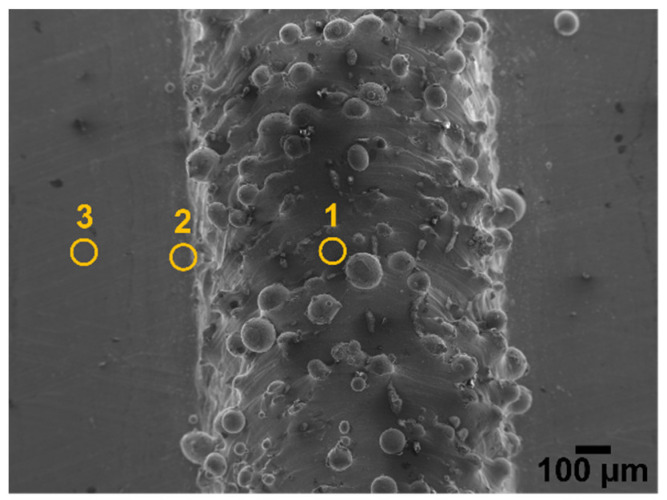
Secondary electron image of the top view of a printed clad in which three regions are marked from where EDX analysis was conducted: (region **1**) on the top of the printed clad, (region **2**) in the close vicinity of the printed clad at the HAZ, and (region **3**) on the base plate relatively far from the printed clad.

**Figure 6:**
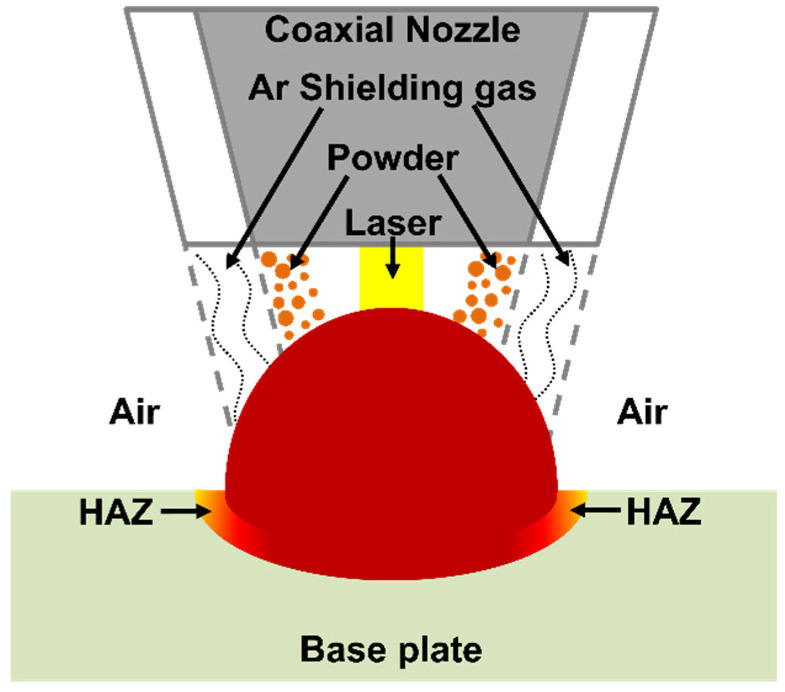
Schematic representation of the coaxial nozzle system with laser and powder flow being surrounded by Ar shielding gas. Portions of the printed clads that are left behind by the moving nozzle during manufacturing and the vicinity of the clads (for instance the HAZ region) are exposed to ambient air while still at elevated temperatures.

Aside from the large depletion of alloying elements, these HAZ regions were characterised by an elevated content of oxygen (between 20 and 30 wt.%), marking a high level of oxidation in the areas next to the deposited clads. An EDX analysis on the top surface of the specimens also revealed a high level of oxidation on the surface of the LMD-deposited clads (from 10 to 25 wt.%). However, in general, in a single clad a higher content of oxygen was always measured on the HAZ compared to the surface of the clad. Figure 5 presents the top view of a printed clad, showing three representative zones where EDX spectra were acquired. The measured chemical composition of the zones highlighted in Figure 5 are shown in Table 2. As can be seen in these representative examples (Figure 5 and Table 2), the surface of the clads is highly oxidised, however, an even higher oxygen content was measured on the HAZ. On the other hand, a very low oxygen content was measured on the base plate relatively far from the printed clad, a content that is typical of surface native oxides. These high levels of oxygen on the clads and in their close vicinity are thermally driven oxides. Even though argon was used as shielding gas, this is only located around the stream of powder ejected from the nozzle, as seen in the schematic of Figure 6. The portions of the printed clads that are left behind by the moving nozzle during manufacturing and their surroundings are exposed to ambient air while still being at elevated temperatures, promoting thermal oxidation. The relatively higher content of oxygen in the HAZ compared to the surface of the clads can be due to the fact that the HAZ is not reached by the stream of shielding argon gas (Figure 6), being exposed to air at all times during the printing. On the other hand, the surface of the clads are temporarily protected by shielding Ar gas and left behind by the moving nozzle only after some cooling has already occurred, being exposed to air at relatively lower temperatures than the HAZ region. 

**Figure 7:**
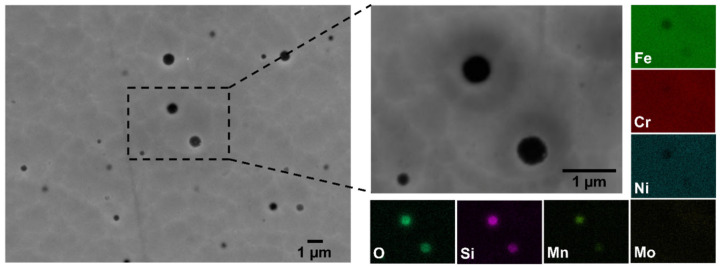
Secondary electron micrographs of regions in DED printed parts made up of several layers in which numerous manganese silicate inclusions can be observed (as measured with EDX analysis). The samples were fabricated with an overlapping of the clads of about 20%.

**Figure 8:**
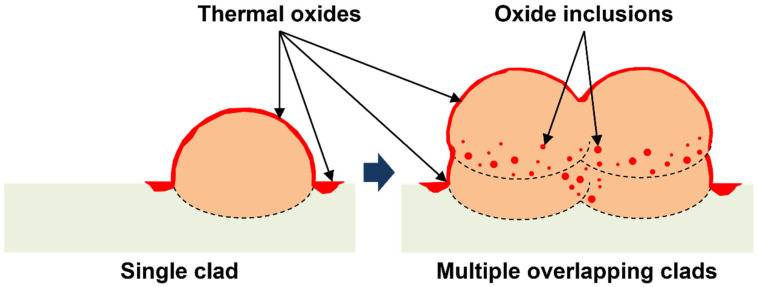
Schematic representing the formation of oxide inclusions from the thermal oxides formed on the HAZ and on the surface of the printed clads when multiple overlapping clads are deposited by DED.

The high content of thermal oxides formed during the DED manufacturing process on the HAZ and on the surface of the printed clads can be the source of the high amount of oxide inclusions often found in multi-layered DED 316L SS [10,30,44,45]. While no inclusions could be found on the deposited single clads analysed, this was not the case when several layers of the material were deposited by DED. Figure 7 shows micrographs of regions in DED printed parts made up of several layers in which numerous manganese silicate inclusions can be observed (as measured with EDX analysis). The schematic of Figure 8 graphically represents the formation of oxide inclusions from the thermal oxides formed on the HAZ and on the surface of the printed clads when multiple overlapping clads are deposited by DED. These oxide inclusions can greatly affect the corrosion performance of the manufactured parts by negatively influencing the passivity of the native oxide layer. However, this is out of the scope of this work since the present study is dedicated to the characterisation and corrosion performance of single clads (the simplest system relevant for repair applications), in which these oxide inclusions could not be found. The investigation of the effect of inclusions on the corrosion behaviour of DED printed parts will be the topic of a future study.

### 3.4. Un-Melted Powder Particles

**Figure 9:**
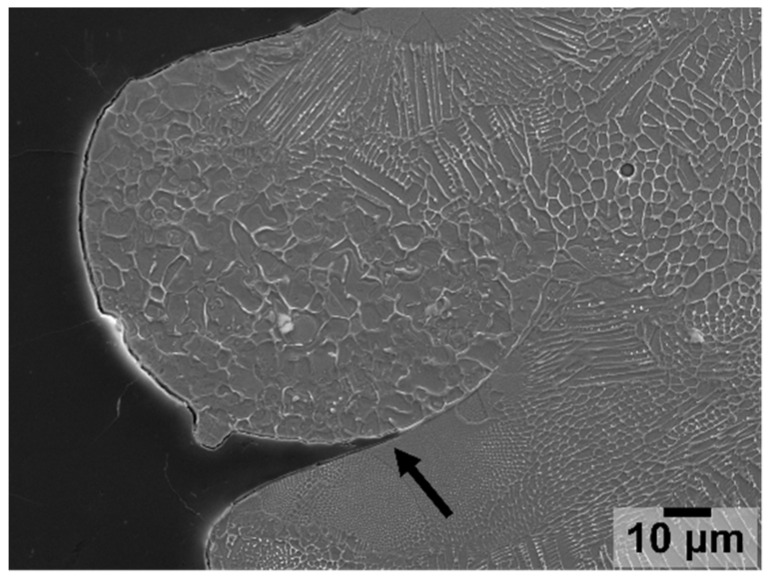
Secondary electron image of a partially un-melted powder particle at the top of the deposited clad.

Partially un-melted powder particles were observed randomly distributed across the surface of the printed clads. Different degrees of fusion between these particles and the deposited clads were observed. While some particles were almost fully fused on the printed clad, others were only fused in part, also presenting regions in which a gap could be identified between the particle and the surface of the clad (see Figure 9). Particles presenting a gap with the LMD-deposited material, such as that represented in Figure 9, were mainly observed on the lateral sides of the clads, and to a much lesser extent on the top surface of the clads. These gaps between the partially un-melted powder particles and the deposited material are of special interest during the analysis of the corrosion behaviour of these samples since these locations are potential hot spots for corrosion initiation in the form of crevice corrosion.

### 3.5. Voids and Pores

**Figure 10:**
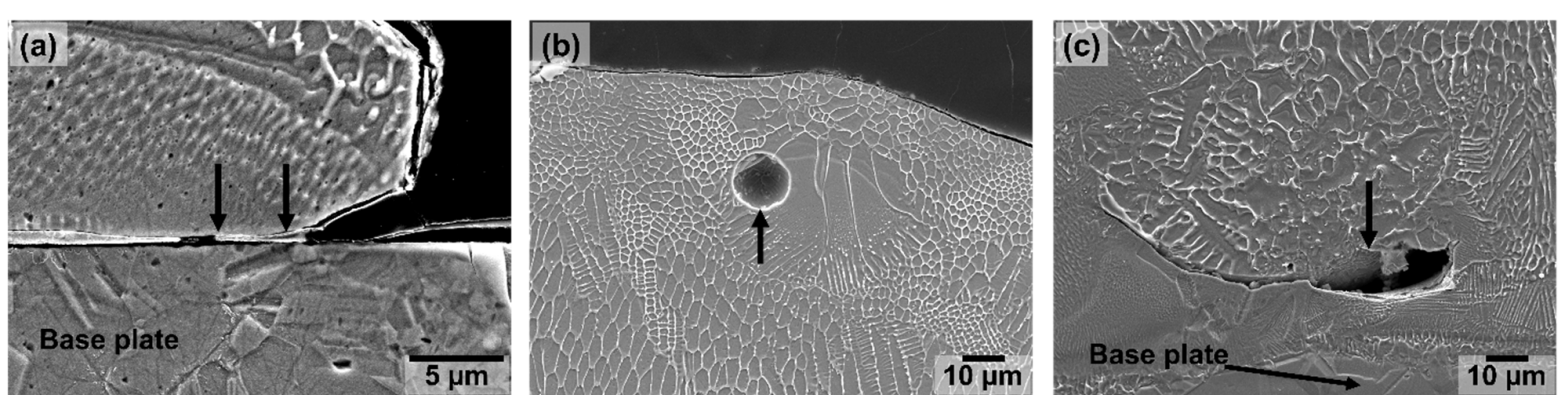
Secondary electron image of (**a**) a region at the border of a deposited clad showing a gap due to insufficient fusion between the deposited clad and the base plate, (**b**) a region in the cross section of the printed clad in which a gas pore is visible, and (**c**) a region around the interface between the printed clad and the base plate in which a lack-of-fusion pore can be seen.

Alongside some gaps between un-melted powder particles and the printed clads, other voids and pores could also be identified during the analysis of the cross sections of the samples (see Figure 10). These voids and pores were seen distributed in a seemingly random fashion on all the samples analysed, independently of the process parameters. One type of void observed was occasional gaps between some regions at the border of the clad and the base plate (see Figure 10a). These gaps are caused by insufficient fusion between the deposited clad and the base plate. They were seen in cross sections of clads prepared with a relatively low energy density, as well as in clads deposited with a relatively high energy density (laser energy input into the material). Therefore, no clear link could be established between the processing condition and the formation of these defects for the range of process parameters analysed. As discussed for the case of gaps between partially un-melted powder particles and the surface of the printed clads, these gaps are also regions of interest since corrosion could potentially initiate there in the form of crevice corrosion due to the direct access of the environment and corrosive media to these voids.

Pores within the deposited material were also observed during the analysis of the cross sections. These pores could be divided into two types as seen in Figure 10b,c. The first type is relatively small (typically bellow 10–20 µm in diameter) with an approximately spherical shape (Figure 10b). These are trapped-gas pores resulting from gas trapped during the metal deposition or from gas previously trapped within the powder particles during gas atomisation. The second type of pores were found at the interface between the deposited clad and the base plate (Figure 10c). These pores were larger in size than the gas pores and had an irregular shape. These are lack-of-fusion pores, which are the result of insufficient fusion between the deposited metal powder and the base material. These types of defects, extensively described in the literature, will play no role in the corrosion performance of these samples during simple exposure of the system to a corrosive media since they are positioned in the interior of the material. Nevertheless, they might play a role in the susceptibility of the printed parts to corrosion fatigue and stress corrosion cracking. However, these issues are out of the scope of this study. In any case, it is important to highlight that the samples analysed presented almost no porosity, and the images portrayed in Figure 10b,c are some of the very few micrographs analysed in which porosity was observed.

### 3.6. Corrosion Behaviour

**Figure 11:**
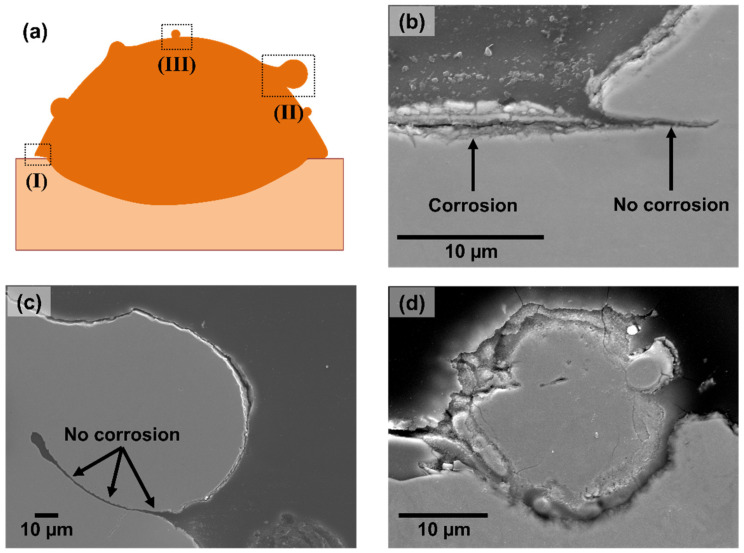
Images of samples after immersion in 3.5 wt.% NaCl for 1 week. (**a**) Schematic of the cross section of a typical printed clad in which specific features are highlighted: I—(**b**) region at the border of the deposited clad in which a gap exists due to insufficient fusion between the deposited clad and the base plate (corrosion is not seen at the gap but rather next to the clad, at the heat-affected zone), II—(**c**) region at the surface of the printed clad with a partially un-melted powder particle of average size or larger (no corrosion was observed around these relatively larger particles), and III—(**d**) region at the surface of the printed clad in which a small, partially un-melted powder particle is visible (corrosion around these small particles was systematically observed).

After immersing the samples in the corrosive media described in the experimental section, cross sections and top views of the deposited clads/base plate were analysed. General inspections of the samples as well as a dedicated examination of the regions of interest (as seen in the schematic in Figure 11a) were carried out. Overall, limited corrosion attack was seen on the samples after one week of immersion. However, this exposure time was enough to clearly identify the main features contributing to the corrosion initiation process.

**Figure 12:**
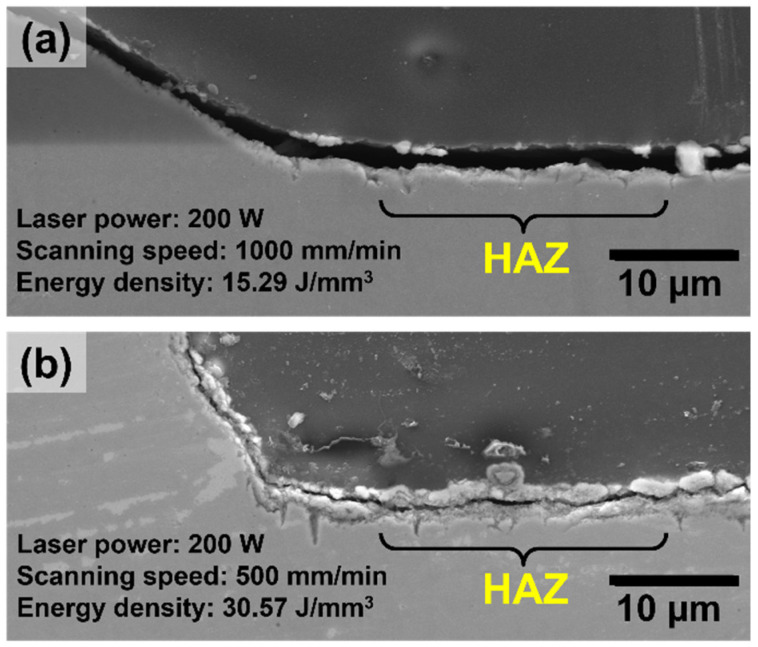
Cross-section secondary electron images of the corrosion morphology on the heat-affected zone of clads deposited using relatively low (**a**) and high (**b**) laser energy density. A much more severe corrosion attack can be seen for clad (**a**) than for clad (**b**).

Figure 11 presents results of corrosion after immersion in 3.5 wt.% NaCl. Most corrosion activity was noticed on the surface of the base plate in close vicinity of the deposited clad, exactly where the heat-affected zone is located. The analysis of regions at the border of the deposited clad, in which a gap exists due to insufficient fusion between the deposited clad and the base plate, showed that these gaps made almost no contribution to the corrosion process, but rather the HAZ was the main region affected (see Figure 11b). The high susceptibility of the HAZ to corrosion attack can be explained by the high depletion of alloying elements (especially Cr and Mo) in these regions, which greatly reduces the pitting resistance of this zone. Furthermore, the EDX analysis carried out on the surface of the HAZ (Figure 4b) showed that the HAZ is mainly composed of Fe oxides, which do not offer much protection against corrosion under the given exposure conditions. Furthermore, a detailed analysis of the corrosion morphology on the HAZ revealed a more severe corrosion attack for samples prepared using a relatively higher energy density. This can be seen in Figure 12, in which two clads deposited using different scanning speeds, and hence different energy densities, are shown. The energy density indicates the laser energy input into the material, and was calculated in this case as the laser power divided by the scanning speed and the area of the laser spot, which had a diameter of 1 mm. The clad presented in Figure 12a was manufactured with an energy input of 15.3 J/mm^3^, while the clad in Figure 12b was fabricated using 30.6 J/mm^3^. The use of a relatively higher energy density can lead to a greater diffusion/depletion of alloying elements in the HAZ and, therefore, to a larger decrease in the PREN of that region.

Additionally, a large number of partially un-melted powder particles on the surface of the printed clads were analysed using SEM. Very little to no corrosion was observed around relatively large particles, and even within the gaps formed between some of these particles and the surface of the deposited clads (see Figure 11c). On the other hand, relatively small powder particles (<30 µm in diameter, representing barely 5% of all the particles within the metal powder) and highly irregular particles were identified to be the main source of corrosion among all the (partially un-melted) particles on the surface of the clads (see Figure 11d). Highly irregular powder particles might be covered by a very defective oxide layer with flaws that can easily promote pitting corrosion initiation. The case of small powder particles being more susceptible to corrosion attack than relatively larger particles on the surface of the deposited clad is very interesting since this observation had never been reported before. The relatively higher susceptibility to corrosion of the small powder particles could be related to an increased surface reactivity with the reduction in the particle diameter. Previous studies have suggested that decreasing the powder particle size greatly increases the surface energy of these particles [46], which could in turn increase their reactivity.

**Figure 13:**
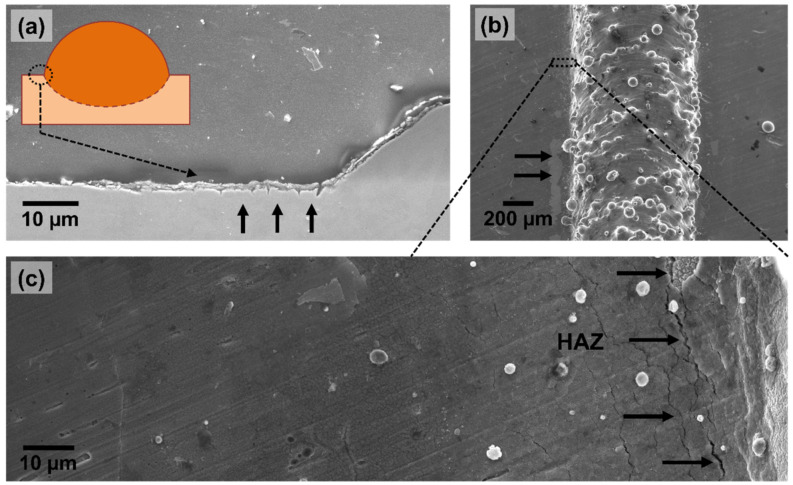
Images of samples after immersion in 3.5 wt.% NaCl at pH 2 for one week. Secondary electron images of (**a**) the cross section and (**b**) the top view of a printed clad. (**c**) High magnification of the region marked in (**b**) around the heat-affected zone.

The corrosion morphology of the samples immersed in acidified 3.5 wt.% NaCl also revealed particularly severe corrosion attacks along the HAZ and on relatively small or highly irregular powder particles partially melted on the surface of the clads. Nevertheless, in this case, some scattered areas on the surface of the clads were also noticed to be affected by slight general corrosion. Under these low-pH conditions, Fe oxides are known to be unstable and easy to dissolve which initiates general corrosion. In any case, as with the samples immersed in the neutral NaCl solution, the heat-affected zones were the most severely affected areas of the samples by the corrosion attack. This is marked by arrows in the cross section and top view images shown in Figure 13. From Figure 13a,c it can be clearly seen that the corrosion on the base plate in the vicinity of the clad does not extend further than 30 to 40 µm from the printed clad, exactly coinciding with the heat-affected zone described in the previous section.

Previous studies have reported a much higher pitting corrosion resistance for additively manufactured 316L SS compared to wrought material due to the presence of a sub-granular cellular network (as described in the previous section) [10,26,27,28] or the effective elimination of inclusions during MAM [23,24]. However, it should be noticed that the corrosion attacks on the systems analysed in this work were mostly driven by defects associated with the LMD process. The role of the sub-granular cellular network seen in the printed clad on the protectiveness of the clad against corrosion was insignificant compared to the role of defects on the corrosion process. It is also important to highlight that the studies considering the microstructure effect on the corrosion behaviour of these materials have been mainly carried out on polished specimens. Therefore, this work highlights the necessity to conduct more investigations on as-built surfaces to better explore the conditions of the materials closer to their final applications. This study also demonstrated that the defects present in this system, which are highly relevant for repair applications, play a predominant role in its corrosion performance. Furthermore, although the depletion of alloying elements in the HAZ is a known issue from conventional welding metallurgy of 316L stainless steel, this work highlights that this is also the main feature affecting the corrosion performance of parts repaired by powder-based DED technology, despite the formation of several other defects intrinsic from the additive manufacturing process.

It is important to highlight that this study is limited to single clads of 316L SS deposited on a flat wrought 316L base plate. The complexity of the system might increase when depositing several overlapping clads or repairing irregular or damaged structures. Overlapping clads will promote the formation of numerous oxide inclusions within the printed part (as seen in Figure 7 and Figure 8) due to the deposition of the clads on top of relatively thick thermal oxides (from previous HAZs and the surface of previously deposited clads), which can also play a role in the corrosion performance of the parts. Overlapping clads will also create a more complex distribution of HAZs with re-heated and crossing/overlapping HAZs. Moreover, repairing irregular/damaged parts might influence the interface between the clads and the repaired base material since the surface of these parts prior to LMD printing could be covered with oxy-hydroxides or corrosion products from environmental exposure. Printing on very rough surfaces can also influence the local position of the laser focus relative to the surface during printing, and consequently affect locally the extent of the HAZ. Furthermore, repairing parts of dissimilar material as the one deposited might also add other factors that can have a great impact in the corrosion behaviour of the repaired pieces. For instance, this could lead to elemental diffusion near the interface, galvanic corrosion phenomena, and/or the formation of other cracks and voids near the interfacial region due to dissimilarities in the thermal properties and solidification process of the joint materials.

Future research will focus on a more detailed characterisation of the interface between the repaired part and the DED-deposited material, systematically analysing the elemental distribution, dislocation density, and grain structure in this region. Localised corrosion analyses will also be carried out (by microcapillary technique) to gain a better understanding of the (independent) electrochemical response of the different features and defects in the repaired system.

### 3.7. Considerations towards Improving Corrosion Performance of LMD 316L SS Parts for Repair Applications

The higher the depletion of alloying elements within the HAZ, the lower the pitting corrosion resistance of this area will be. Corrosion attacks along the HAZ, located adjacent to the deposited material, could compromise the integrity of the printed/repaired part and therefore of the entire system. Therefore, minimising the extension of the HAZ as well as the level of depletion of alloying elements is of paramount importance to increase the corrosion resistance and improve the performance of LMD 316L SS parts deposited on wrought material for repair applications. Further research should focus on systematically analysing the relationship between additive manufacturing process conditions and the structure/morphology and composition of the heat-affected zone.

Moreover, since relatively small powder particles contributed actively to the corrosion process, the use of metal powder with a relatively large particle size is recommended from a corrosion perspective. Furthermore, the use of surface finishing techniques, such as shot pinning, sand blasting, electropolishing, or laser re-melting, to reduce/eliminate the partially un-melted powder particles remaining on the surface of the printed part should also be explored. 

## 4. Conclusions

This work characterised the different defects and microstructural features formed in 316L stainless steel clads deposited on wrought material by laser-based DED, highlighting their role in the corrosion performance of this system. The main conclusions of the study can be summarised as follows:

1. Heat-affected zones along the deposited clads (in their close vicinity) were characterised by a large depletion of alloying elements, while containing a high oxygen content due to thermally driven oxidation. Depletion of Cr and Mo in these regions provoked a significant decrease in the pitting resistance of the heat-affected zone, resulting in these regions being the primary contributor to the corrosion activity of the LMD-deposited clads.
2. Samples prepared using a relatively higher laser energy density presented a more severe corrosion attack along the heat-affected zone than clads manufactured with relatively low laser energy input.
3. From the partially un-melted powder particles scattered across the surface of the deposited clads, almost exclusively powder particles with a relatively small diameter (generally <30 µm) and a highly irregular shape were actively affected by corrosion.
4. Due to the exposure to ambient air of the HAZ and the surface of the printed clad left behind by the moving nozzle, a relatively high level of thermal oxidation was observed on these regions. These high contents of thermal oxides formed during the DED manufacturing process on the HAZ and on the surface of the printed clads can be the source of the high number of oxide inclusions often found in multi-layered DED 316L stainless steel parts.
5. This work highlights the need for more investigations on as-built surfaces of additively manufactured parts to better understand the performance of the materials closer to their final applications, demonstrating that the surface defects resulting from the additive manufacturing process play a predominant role in the corrosion behaviour of the system.

## Figures and Tables

**Table 1:** Composition of 316L stainless steel in wt.% with balance of Fe.

Cr	Ni	Mo	Mn	Si	S	C	N	P
16.0–18.0	10.0–14.0	2.0–3.0	<2.0	<0.75	<0.03	<0.03	<0.1	<0.05

**Table 2:** Elemental composition calculated from the EDX spectra acquired in the regions highlighted in Figure 5.

	Wt.%							
	Fe	O	Cr	Ni	Mo	Si	Mn	S
Spectrum 1	53.9	15.6	16.3	10.2	2.5	0.5	1	–
Spectrum 2	57.1	21.2	13.6	5.3	0.9	0.3	1.4	0.1
Spectrum 3	68.2	1.3	16.6	10.0	2.0	0.5	1.5	–

## Data Availability

The raw data required to reproduce these findings can be made available upon reasonable request.
